# Welfare of calves and heifers on dairy farms with cow-calf contact rearing or early separation

**DOI:** 10.3389/fvets.2025.1610084

**Published:** 2025-08-08

**Authors:** Anna Rademann, Marie Louise Schneider, Susanne Waiblinger

**Affiliations:** Centre for Animal Nutrition and Welfare, Clinical Department for Farm Animals and Food System Science, University of Veterinary Medicine Vienna, Vienna, Austria

**Keywords:** cattle, dam-calf contact, dairy calves, dairy heifers, behavior, health, welfare assessment, foster cow

## Abstract

Early separation (ES) of cow and calf in dairy farming is increasingly questioned due to implications on animal welfare. The aim of this study was to compare the welfare of animals on commercial dairy farms with cow-calf contact (CCC) or ES using a comprehensive welfare assessment protocol. We hypothesized that the welfare of calves and heifers on CCC farms is better than the welfare of those on ES farms. Fifty Austrian dairy farms, 25 practicing CCC and 25 ES, were visited. The Welfare Quality® (WQ) Protocol for dairy calves and heifers was used to assess animal welfare. The two rearing systems were compared using a t-test for qualitative behavior assessment (QBA) scores, a Mann–Whitney U Test for quantitative behavioral indicators, prevalences of clinical scoring, management parameters, and Criterion and Principle scores, and a Fisher Exact Test for dichotomous variables (occurrence yes/no) and overall classification. CCC calves and heifers scored higher in QBA (calves: *p* < 0.001, heifers: *p* = 0.022). CCC calves showed a lower frequency of non-nutritive oral behaviors (*p* = 0.038). Both CCC calves and heifers had more space (calves and heifers: *p* < 0.001), were less often disbudded (calves: *p* = 0.002, heifers: *p* = 0.003) and had more access to pasture (*p* < 0.001). Fewer CCC farms had calves with lesions (*p* = 0.049) and heifers with overgrown claws (*p* = 0.017). Accordingly, rearing systems differed in Criterion and Principle scores. Both CCC calves (*p* = 0.011) and heifers (*p* = 0.043) scored higher in “Appropriate Behavior” and calves scored higher in “Good feeding” (*p* = 0.047) and “Good housing” (*p* = 0.001). CCC farms had a better WQ classification than ES farms for calves (*p* = 0.023), and 20% or 26% of CCC farms reached “excellent” for calves or heifers compared to 0 or 12%, respectively . The better welfare of CCC animals may be related to contact to cow(s) but also to other management practices that emphasize animals’ physical and behavioral needs more. Holistic practices prioritizing animal welfare including CCC systems should be considered in the transition toward more sustainable farming.

## 1 Introduction

Cattle are highly social animals with complex social, emotional and cognitive characteristics (1). Although it opposes their natural behavior of establishing strong mother-filial bonds, cows and calves are routinely separated shortly after birth (2, 3). Besides evidence of public concern for early separation (ES) of cow and calf (4), also scientists have been calling for a change in the dairy production system [e.g., (5)] due to various harmful effects of ES on both cow and calf and benefits of keeping them together for a prolonged time [reviewed in Johnsen et al. (6), Beaver et al. (7), Meagher et al. (8)]. For example, calves reared without contact to their dam frequently show abnormal oral behavior like cross-sucking or object licking (9, 10) that can become chronic (11), whereas in cow-calf contact (CCC) systems, such behavior is absent or strongly reduced (2, 12, 13). In calves reared with contact to their dam, growth rates during the suckling period are higher compared to artificially reared calves (13, 14). Calves grow better when kept with their dam the days after birth even if suckling is prevented (15), which suggests beneficial effects of dam-rearing beyond nutritional effects, potentially mediated by beneficial effects of higher oxytocin levels (16, 17) and lower plasma cortisol concentrations (18). Moreover, CCC calves played more than ES calves (19), indicative of a more positive emotional state (20). Also beneficial effects of CCC on cow and calf health have been observed [e.g., (12, 21)], although some studies found contradictory effects [(22); reviewed in Beaver et al. (6)]. Moreover, CCC animals develop increased social competence and sociality as calves and as heifers (23–26). In terms of the animal-human relationship (AHR), CCC calves may be at higher risk of developing a poorer AHR, as no regular contact with humans during feeding is necessary, but results of experimental studies comparing ES and CCC are contradictory (27–29).

Even if CCC systems seem to have mainly positive effects on animal welfare compared to ES, data on welfare implications, especially under commercial conditions, is still limited (2). Moreover, despite some evidence on better growing and performance rates of heifers in an experimental setting (30), the welfare of heifers on CCC farms compared to ES has not yet been studied comprehensively. Besides the effects of CCC per se, CCC farms may also differ from ES farms in other management practices affecting animal welfare, such as performing disbudding or provision of pasture access, because allowing their animals to perform more natural, species-specific behavior is an important value for CCC farmers (31, 32).

Assessing animal welfare is complex and requires comprehensive approaches, considering mainly animal-based indicators covering the different welfare dimensions and the whole range of the welfare continuum from poorest to best (33–35). To create an internationally standardized, open-access tool based on scientific evidence that meets the above-mentioned needs as well as considering the public demand for sufficient protection of farmed animals (36) and the importance of animal welfare in the context of sustainability (37, 38), European scientists developed the Welfare Quality® Protocol (39, 40). The WQP is an established tool that has been used repeatedly for dairy cows [e.g., (41)] and fattening bulls (42) to assess animal welfare based on multiple measures that can be integrated to Criterion and Principle scores and an overall classification of the farm. However, to our knowledge, there has not yet been a study using the WQP for dairy calves and heifers (43), nor has the WQP been used to assess the welfare of animals in CCC systems on-farm.

Thus, we aimed to investigate possible differences in the welfare of calves and heifers on CCC farms as an innovative system compared to ES farms as a reference system in a comprehensive approach using WQP. The data was collected as part of the project “COwLEARNING for sustainable beef and dairy supply” that, *i.a.*, aims for a comprehensive farm-to-fork sustainability assessment using the present data for an integration of animal welfare as one dimension of sustainability (44, 45).

We hypothesized that animals on CCC farms would experience better welfare in terms of (i) a more positive affective state, reflected in higher scores for Qualitative Behavior Assessment (QBA) and more play behavior, (ii) lower occurrence of abnormal oral behaviors, (iii) better health, and (iv) beneficial effects of improved management, i.e., less disbudding and more access to pasture. No effects of rearing system on the avoidance distance of the animals were expected. Considering this, we hypothesized that overall welfare would be better in animals on CCC farms, reflected in higher WQP scores and better WQP classification for those farms. As we expected calves to be more vulnerable to the absence of contact to a cow, greater differences in the welfare of calves between CCC and ES farms than in heifers were expected.

## 2 Animals, material and methods

### 2.1 Farms and study design

We compared two cohorts of, in total, 50 dairy farms across Austria, 25 farms practicing CCC and 25 ES. A convenience sample of CCC farms was selected based on herd size (it was aimed at 15–60 cows) and distribution across federal states and geographical regions. Farms with ES were selected accordingly to optimally balance herd size and region between the two rearing systems. The farm selection criteria included that cows were housed in a loose-housing system, i.e., housed not in tie-stalls, but in deep litter, compost or free stall barns, and that the conditions on the farm (e.g., calf rearing system and type of barn) needed to be in place for at least 2 years to (i) avoid to detect aspects related to changing the system and not the system per se and (ii) have one full financial year available for economic calculations in the framework of the farm-to-fork sustainability assessment (not part of this paper).

Farmers were recruited by contacting them personally from mailing lists from former projects asking for participation, as well as by sharing information about the project via local and national farmers’ associations through flyers and/or personal communication to which farmers interested in participation contacted the researchers. Farmers received feedback including anonymous benchmark results after the visits.

A farm was defined as practicing CCC when calves were allowed to suckle their dam or a foster cow for at least 12 weeks. However, to reach the desired sample size, two farms where cow and calf were separated at 4 weeks or between 3 and 8 weeks after birth, respectively, were included as CCC farm. Farms with dam-calf-contact, foster cow and mixed rearing were included as well as farms with different durations of daily contact [i.e., CCC twice a day, half a day or whole-day; see (46), for terminology]. Farms were defined as ES when cow and calf were permanently separated at maximum 24 h after birth.

### 2.2 Data collection

Animal welfare was assessed using the WQP for dairy calves and heifers (43). Cow welfare was also assessed using the WQP for dairy cows (47), however the results will be presented in a different paper (Schneider et al. in preparation). The WQP is based on the 4 principles “Good feeding,” “Good housing,” “Good health” and “Appropriate Behavior.” These principles comprise 12 criteria, each based on one to several measures (Table 1). Data were recorded on paper except for clinical parameters that were entered digitally on a tablet using Microsoft Lists version 2,309 (48).

**Table 1:** On-farm measures and their assignment to criteria and principles according to the WQP for dairy calves and heifers (43).

Principle	Criterion	Measure
Good feeding	Absence of prolonged hunger	% of thin animals
	Absence of prolonged thirst	Number, functioning and cleanliness of drinkers
Good housing	Comfort around resting	% of dirty animals
		*Mean duration of lying down* ^1^
	Ease of movement	Available space per animal, access to exercise yard
Good health	Absence of injuries	% animals with hairless patches and/or lesions and/or swellings
		% animals with overgrown claws^1^
		% lame animals
	Absence of disease	Number of coughs/animal/15 min
		*Number of sneezing/animal/15 min*
		Hampered respiration
		% animals with nasal discharge
		% animals with ocular discharge
		% animals with diarrhea
		*% animals with umbilic infection^2^*
		% animals with bloated rumen
		*% animals with ear infection*
		% mortality in the last 12 months
	Absence of pain induced by management procedures	% animals disbudded/dehorned including use of anesthetics and/or analgesics
		% of animals tail-docked including use of anesthetics and/or analgesics
Appropriate behavior	Expression of social behaviors	Agonistic interactions (headbutt, displacement, *fighting, chasing, chasing up*)/animal/h
		Social affiliative behavior (social licking, *horning*)/animal/h
	*Absence of abnormal behavior*	*Non-nutritive oral behaviors (tongue rolling, object licking, inter-sucking and urine drinking)*/animal/h
	Good human-animal relationship	Avoidance distance test
	Other behaviors	Days with > 6 h access to pasture
	Positive emotional state	*Play bouts/animal/h*
		Qualitative behavior assessment

Measures and criteria written in italics were recorded and analyzed, but not included in calculations for WQ Scores. ^1^This measure is not observed in calves. ^2^This measure is not observed in heifers.

Farm visits took place from February till April 2023 and from October 2023 till April 2024. Farms were only visited outside of pasture season to minimize seasonal effects and have comparable conditions between farms with and without pasture access. Although only visited during winter, the possibility for access to pasture in the summer is considered in the calculation of WQ scores for appropriate behavior.

Each farm was visited for 1 day. Assessments always started directly after morning feeding with assessing AD, followed by QBA, quantitative behavioral observations and clinical scorings (43). The time of assessment of management conditions varied, however, it was always carried out after behavioral observations to avoid variations in behavior due to time of day. Calves were defined, according to EU Council Directive 2008/119/EC, as cattle up to 6 months. Heifers were all female cattle > 6 months until first calving (43).

#### 2.2.1 Behavior

##### 2.2.1.1 Avoidance distance (AD)

Avoidance distance (50, 51) was aimed to be tested at the feeding place. To conduct the test, the animal needed to have its head completely outside the feeding rack. The experimenter placed herself at a distance of at least 2 m in front of the animal. After ensuring that the animal noticed the presence of the experimenter, the latter slowly approached the animal with a pace of one step per second with her arm held outstretched toward the animal in an angle of approximately 45° in front of the body. The experimenter walked toward the animal until signs of withdrawal (i.e., the animal moves backwards, turns the head to the side or pulls it back to exit the feeding rack) or until touching of the nose or muzzle. In the case of withdrawal, the AD is the estimated distance between hand and muzzle at the moment of withdrawal in 10 cm resolution. If the animal could be touched, the AD was noted down as 0. If the animals were single-housed, the limited ability to withdraw due to restricted space was noted down. When it was not possible to conduct the AD tests at the feeding place, e.g., due to calves being housed without a feeding rack, AD was tested in the barn. Every animal was tested once.

##### 2.2.1.2 Qualitative behavior assessment

Duration of QBA observation was 15 min for calves and heifers each. Observations were always carried out from outside the pen. The expression of 20 pre-defined adjectives, including, e.g., active, relaxed, tense, enjoying, happy, and distressed, in relation to the observed group was scored on a 125 mm scale (0 = not present at all, 125 = highest imaginable expression). The values were then turned into an index using a weighted sum with weights for each term and calculation instructions specified in the WQP [for details see 2.4 and Welfare Quality® Network (52)].

##### 2.2.1.3 Quantitative behavior observations

Behavior was observed quantitatively for 60 min for calves and heifers each. Duration was equally split between calves aged 0–2 and 3–6 months and heifers aged 7–12 and > 12 months, i.e., the behavior of each age category was observed for 30 min, leading to a total observation time of 120 min across the four groups (43). Behavior was observed continuously. The maximum group size that was observed at the same time was 20 animals, and observations were always carried out from outside the pen. If the group was bigger or there were several groups within an age category, more than one observation point was defined, and the total observation time was split among the observation points for each category. The assessors only started the observation a few minutes after taking the position at the observation point to minimize influences on the animals’ behavior resulting from their presence. At the beginning of each observation, the number of animals lying, feeding/drinking and standing was noted down. The ethogram can be found in Table 2.

**Table 2:** Ethogram of the WQP for dairy calves and heifers (43).

Behavior	Definition
Tongue rolling	An animal repeatedly twisting, twirling, and/or swinging the tongue inside or outside the open mouth, or stretching the tongue for longer than 5 s, sometimes with neck and head stretched somewhat upwards. New bout counted after a pause of ≥ 10 s
Object licking	An animal chewing or licking any non-edible equipment. New bout counted after a pause of ≥ 10 s
Cross-sucking	The actor pulling on teat, udder, ear, tail, or skin of a group mate with the muscles of her/his cheeks and tongue for longer than 5 s. New bout counted after a pause of ≥ 10 s or if receiver changed
Urine drinking	An animal drinking the urine or sucking the prepuce of another animal
Head butt	Interaction involving physical contact: the actor is butting, hitting, thrusting, striking or pushing the receiver with forehead, horns or horn base with a forceful movement; the receiver does not give up its present position
Displacement	Interaction involving physical contact: the actor is butting, hitting, thrusting, striking or pushing the receiver with forehead, horns, horn base or any other part of the body with a forceful movement. As a result, the receiver gives up its position. Includes an animal shoving itself between two others or between an animal and barn equipment
Chasing	Actor making another animal flee by following fast or running behind it (only recorded if it follows an interaction with physical contact)
Fighting	Two contestants vigorously pushing their heads (foreheads, horn bases and/or horns) against each other while stemming their feet into the ground in sawbuck position and both exerting force against each other
Chasing-up	The actor uses forceful physical contact (e.g., butting, pushing, shoving) against a lying animal which makes the receiver rise
Social Licking	The actor touches with its tongue any part of the body (head, neck, torso, legs, tail) of another group mate except for the anal region or the prepuce. New bout counted after a pause of ≥ 10 s or if receiver changed
Horning	Animals rubbing their foreheads, horn bases or horns against head or neck of one another without obvious agonistic intention. None of the animals takes advantage to become a victor. New bout counted after a pause ≥ 10 s or if partner changed
Locomotor play	The animal runs and/or comes with 2 or more legs off the ground (run/jump behavior), but no mounting
Mounting	An animal lifting itself up on its hind legs and jumps with its forelegs onto another group mate either from behind, the side or front
Falling	An animal accidentally losing balance, its body quickly moves toward the ground and touches it with udder, sternum, carpal joint, knee or with the whole side or abdomen
Sneezing	Convulsive expulsion of air from the nose
Coughing	Sudden and noisy expulsion of air from the lungs through the mouth
Duration of lying down	Time recording starts when one carpal joint of the animal is bent and lowered (before touching the ground) and ends when the hind quarter of the animal has fallen down and the animal has pulled the front leg out from underneath the body

#### 2.2.2 Physical parameters

Evaluation of physical parameters was carried out for every animal. For each animal, one side of the body as well as the opposite hind leg was randomly chosen for examination. Both sides were approximately equally represented among each herd. Definitions for each parameter can be found in Table 3.

**Table 3:** Definitions of measurements of physical indicators according to the WQP for dairy calves and heifers (43).

Indicator	Definition
Body Condition Score	“Too lean” when the body mass of at least three of the regions tail head, loin, vertebrae or “general” (i.e., tail head, hip bones, spine and ribs) was visibly reduced
Lameness	Reluctance to bear weight on one limb or a reluctance to walk
Cleanliness	“Dirty” when at least 25% of the observed side of the animal was covered with plaques or if more than 50% of the area was dirty
Hairless patches	Area with hair loss or extensively thinned hair as a response to parasites, skin not damaged, hyperkeratosis possible; minimum diameter of 2 cm at the largest extent
Lesions/Swellings	Area with damaged skin either in form of a scab or a wound, dermatitis due to ectoparasites as well as overt swelling of a minimum diameter of 2 cm at the largest extent, except for swelling of navel/umbilicus
Overgrown claws	When two of the criteria of a normal claw (plane surface, claw not bended, same length of both claws of the same leg, no or little space between claws, contact to surface of the whole claw, angle to ground near 50°) were not fulfilled in at least one claw
Nasal discharge	Clearly visible flow/ discharge from the nostrils; transparent to opaque (white/yellow/green), often of thick consistency
Hampered respiration	Abnormal breathing, i.e., severe increase of frequency (speed) and intensity (depth)
Ocular discharge	Mucus traces (opaque or abundant transparent secretion or dry accumulation) at the eyes
Ear infection	Animal shows hanging ear or leaning head
Bloated rumen	Belly severely rounded either on the top, below or both
Diarrhea	Accumulation of manure (wet or dry) around tail head
Umbilic infection	Severe swelling of the navel

For hairless patches, lesions, and swellings, the number of alterations per animal was recorded. In case of more than 20 alterations per category or if the total area affected was at least as large as the area of a hand, the maximum number, i.e., “21,” was noted. The other physical indicators were rated for their presence (0 = not present, 2 = present). The farmer was asked about the number of calves that were born and had died or were euthanized in the year before the farm visit to calculate the mortality rate.

#### 2.2.3 Resource and management-based measures

The number (absolute and per animal), functioning and cleanliness of drinkers and material and condition of bedding were assessed for each pen. Available space (m^2^) was measured using a laser distance measuring device. The farmer was interviewed regarding the number of days and hours per day the animals had access to pasture, and management practices such as tail docking, disbudding/dehorning including the use of anesthetics and analgesics, and frequency of feeding and cleaning.

#### 2.2.4 Observers and training

Observations for calves and heifers were carried out by three assessors (assessor 1: 10 farms = 20%, 5 CCC and 5 ES farms; assessor 2: 38 farms = 76%, 18 CCC and 20 ES farms; assessor 3: 2 farms = 4%, both CCC farms). The assessors were trained by a delegate of the Welfare Quality® (WQ) network for 2 days and conducted additional on-farm visits for training purposes prior to the farm visits. Assessment of AD in different locations (at the feeding place, in the barn) was trained additionally by an expert in this field.

### 2.3 Calculation of welfare quality scores

Welfare Quality® Protocols for most animal categories include calculations for the integration of the measures to Criterion and for Criterion to Principle scores. Each score can range from 0 to 100, with 0 being the worst and 100 the best possible welfare. Based on the Principle scores, each farm can be assigned to one of the 4 categories “not classified,” “acceptable,” “enhanced or “excellent” [see Table 1 for assignment of measures to Criteria and Principles; see Welfare Quality® Network (52), for a full description].

The WQP for dairy calves and heifers so far does not include calculations for WQ scores. As developing specific calculations requires capacities that could not be provided within the scope of this project, it was agreed after discussions with members of the WQ Network to use the calculations from the protocol for fattening bulls (52) as most of the indicators proposed in the WQP for dairy calves and heifers are also used in the one for fattening bulls. The data collected on-farm was transferred to excel sheets that were also used to calculate Criterion and Principle scores and the overall classification according to the formulae provided in the WQP for fattening bulls, with some modifications described as follows.

The calculation of the score for the Criterion “Comfort around resting” was modified for both calves and heifers as the measure “Duration of lying down” is not considered a valid indicator according to the WQP for calves (43), and data for this measure was only available for 23 out of 44 farms for heifers. After a discussion with a delegate of the WQ network, it was agreed to replace the partial score for “Duration of lying down” with the following values based on evidence of cattle’s preference for and signs of discomfort or injuries on different lying surfaces [(53–55); reviewed in Tucker et al. (56)]: 0 if animals were lying on concrete (e.g., fully slatted floors), 30 for hard rubber mats, 40 for a completely wet lying surface, 50 for softer cow mattresses, 60 for insufficient straw bedding (e.g., partly dirty) and 100 for sufficient straw bedding. The calculation of the partial score “% of dirty animals” and the weighing of the two scores with a Choquet integral to the Criterion score as described in the WQP for fattening bulls were not changed. For single-housed calves, access to one clean water point was considered sufficient to score 100 for the Criterion “Absence of prolonged thirst,” as opposed to the requirement of at least 2 drinkers available for a group-housed animal.

According to the WQP for fattening bulls, the score for the Criterion “Ease of movement” is determined by the measure “space allowance” [m^2^/700 kg]. This needed to be adapted for calves, as any calf housed under conditions of the minimum of the Austrian Animal Welfare Law would have exceeded the highest score because of their low body weight. After a discussion with a member of the WQ network, it was agreed to keep the general structure of the spline function that is used in the WQP for fattening bulls but to adapt the limit values. It was decided to set the maximum (i.e., Score 100) space allowance to 6 m^2^, as that equals around 3 times the Austrian legal minimum requirements, (i.e., 1.8 m^2^ for calves < 220 kg), a similar proportion as in the WQP for fattening bulls (legal minimum: 3 m^2^/animal > 650 kg, maximum WQP: 9 m^2^/700 kg) and allowing calves at least some forms of locomotor play (2). The minimum (i.e., Score 0) space allowance was set to 0.5 m^2^ for calves aged 0–2 months and 0.7 m^2^ for calves aged 3–6 months so that 20 points, i.e., the threshold to classify as “acceptable” that is considered as minimal requirement, were achieved if the Austrian national legal minimum standards were met [1.6 m^2^/animal for calves < 150 kg, 1.8 m^2^/animal for calves < 220 kg, (57)].

The following measures were not included in the calculation of WQ scores as they are not observed in fattening bulls and, therefore, not included in the formulae: tongue rolling, object licking, cross-sucking, urine drinking, play, mounting, falling, sneezing, and umbilical infection. Still, these parameters were analyzed for differences between CCC and ES farms on measure level.

### 2.4 Statistical analysis

Statistical analysis was performed using IBM SPSS Statistics Version 29.0.2.0 (58). All the measures except for AD were analyzed on farm level. Behaviors were grouped into agonistic (headbutt, displacement, fighting, chasing, chasing up), non-nutritive oral behaviors (tongue rolling, object licking, cross-sucking, urine drinking), cohesive (horning, social licking), play (mounting, locomotor play) and health related (coughing, sneezing, falling). For physical measures with high non-occurrence, i.e., a prevalence = 0 at ≥ 30 farms, the variable was dichotomized, i.e., the occurrence of the measure on the farm (yes/no) was compared between CCC and ES farms. This was the case for lean animals (occurrence on 5 farms for calves, 9 farms for heifers), dirtiness (8/20), lesions (7/15), swellings (3/11), nasal (12/12) and ocular (5/5) discharge and lameness (1/2) in calves and heifers and for diarrhea (9) in heifers (Table 4). All the visited farms were included in each analysis, i.e., for calves: CCC: *n* = 25, ES = *n* = 25; for heifers: CCC: *n* = 19, ES: *n* = 25.

**Table 4:** Results for physical and resource/management measures.

	**CCC Calves** n = 25 farms	**ES Calves** n = 25 farms		**CCC Heifers** n = 19 farms	**ES Heifers** n = 25 farms	
**Physical parameters** ^**1**^ **[%]**	median, range (no. farms with prevalence > 0)	median, range (no. farms with prevalence > 0)	*Mann-Whitney U Test*:**p, z**	median, range (no. farms with prevalence > 0)	median, range (no. farms with prevalence > 0)	*Mann-Whitney U Test*:**p, z**
Hairless patches	0, 0–71 (12)	10, 0–63 (13)	0.828, 0.218	17, 0–87 (18)	22, 0–67 (21)	0.529, 0.629
Diarrhoea	0, 0–60 (12)	10, 0–60 (15)	0.302, 1.031	> 0 on 20 farms or less, therefore listed below		
Overgrown claws	This measure is not observed in calves			0, 0–30 (5)	4, 0–42 (16)	**0.031,** 2.151
Mortality (last 12 months)	0, 0–24 (11)	0, 0–26 (12)	0.627, 0.486	> 0 on 20 farms or less, therefore listed below		
***Physical parameters*** ^***2***^ ***[%]***	median, range (no. farms with prevalence > 0)	median, range (no. farms with prevalence > 0)	*Fisher Exact Test*:**p; OR (95% CI)**	median, range (no. farms with prevalence > 0)	median, range (no. farms with prevalence > 0)	*Fisher Exact Test*: **p; OR (95% CI)**
*Lean animals*	0, 0–33 (2)	0, 0–25 (3)	1; 0.67 (0.12–3.65)	0, 0–14 (5)	0, 0–10 (4)	0.467; 1.86 (0.41–8.22)
*Dirtiness*	0, 0–50 (3)	0, 0–33 (5)	0.702; 0.60 (0.16–2.25)	0, 0–75 (8)	0, 0–84 (12)	0.766; 0.78 (0.24–2.62)
*Lesions*	0, 0–7 (1)	0, 0–33 (6)	**0.049;** 0.14 (0.02–1.01)	0, 0–21 (6)	0, 0–58 (9)	1; 0.82 (0.23–2.91)
*Swellings*	0, 0 (0)^3^	0, 0–33 (3)^3^	4	0, 0–21 (5)	0, 0–58 (6)	0.759; 1.24 (0.35–4.35)
*Nasal discharge*	0, 0–17 (7)	0, 0–33 (5)	0.742; 1.40 (0.51–3.82)	0, 0–19 (6)	0, 0–50 (6)	0.735; 1.46 (0.39–5.55)
*Ocular discharge*	0, 0–10 (1)	0, 0–26 (4)	0.349; 0.25 (0.03–2.08)	0, 0 (0)	0, 0–11 (5)	0.060; –
*Diarrhoea*	> 0 on more than 20 farms, therefore listed above			0, 0–23 (5)	0, 0–10 (4)	0.467; 1.88 (0.41–8.22)
*Hampered respiration*	0, 0–11 (1)	0, 0 (0)	4	0, 0–3 (1)	0, 0 (0)	4
*Lameness*	0, 0 (0)	0, 0 (0)	4	0, 0–8 (1)	0, 0–13 (1)	4
*Bloated rumen*	0, 0 (0)	0, 0 (0)	4	0, 0 (0)	0, 0–4 (1)	4
*Mortality (last 12 months)*	> 0 on more than 20 farms, therefore listed above			0, 0 (0)	0, 0–4 (2)	4
**Resource & Management**	median, range	median, range	*Mann-Whitney U Test*:**p, z**	median, range	median, range	*Mann-Whitney U Test*:**p, z**
Min. space per animal^5^	8.06, 1.35–21.32	2.67, 1.17–11.39	**<0.001,** 3.949	18.3, 3.8–150	8.8, 3.6–41.4	**<0.001**, 3.234
No. days > 6 h on pasture	103, 0–224	0, 0–138	**<0.001**, 3.536	192, 150–356	121, 0–210	**<0.001**, 4.454
*No. days > 6h on pasture (only organic)*	103, 0–224	61, 0–138	**0.015**, 2.431	192, 150–356	150, 89–210	**0.002**, 3.040
**Resource & Management**	No. farms	No. farms	*Fisher Exact Test*: **p; OR (95% CI)**	No. farms	No. farms	*Fisher Exact Test*: **p; OR (95% CI)**
Min. 2 clean, functioning water points yes/no	8/17	2/23	0.074; 5.41 (0.04–0.98)	6/13	5 / 20	1; 1.26 (0.33–4.84)
Disbuddingyes/no	12/13	22/3	**0.005**; 7.94 (1.88–33.50)	8/11	22 / 3	**0.003**; 10.08 (2.22–45.71)

1 Physical parameters with prevalences > 0 on more than 20 farms. ^2^Physical parameters with prevalences > 0 on 20 farms or less. ^3^For swellings, values for calves from 2 CCC and 2 ES farms were not available. ^4^Due to occurrence on less than 4 farms, no statistical tests were conducted for these measures. ^5^For calves: m2/animal, for heifers: m2/700 kg. ^6^For comparison of only organic farms: CCC: *n* = 25 (calves) or *n* = 19 (heifers), ES: *n* = 14 (calves and heifers).

In the published article, there was an error in Table 10 as published. The descriptive and statistical values for Absence of prolonged thirst (only ES calves and CCC heifers), Comfort around resting (only calves), ease of movement (only CCC heifers), Absence of injuries (only ES calves), Absence of disease, Absence of pain induced by management (for both CCC and ES calves and heifers), Good feeding (only ES calves), Good housing (CCC and ES calves and ES heifers) and Good health (both CCC and ES calves and heifers) slightly changed. This is 1) due to the detected changes for the number of clean, functioning water points and disbudding and 2) due to slight necessary corrections for the calculation procedures for ease of movement, absence of injuries and absence of disease that were not displayed correctly in the submitted version. There is now a significant difference for the Criterion score for “Ease of movement” in heifers. However, this does not change the scientific conclusions as the underlying measure “space allowance” had shown a significant difference before and is already discussed in the published manuscript. In addition, there is now a significant difference for “Good feeding” in calves. Also here, this does not change the scientific conclusion as the significant difference in the underlying measure “Number of clean, functioning water points” has been thoroughly discussed in the submitted version. The corrected Table 10 and its caption appear below.

Measures as well as Criterion and Principle scores were first analyzed descriptively and checked for normal distribution using the Shapiro–Wilk Test and Q-Q-Plots. As we did not test multiple influencing factors but aimed for a comparison of the welfare of animals between CCC and ES farms, univariate tests were used, except for AD (see below for an explanation). Only QBA was normally distributed. Therefore, a t-test was used for QBA, Mann–Whitney U Tests for behaviors, prevalences of physical parameters, management parameters, Criterion and Principle scores and a Fisher Exact Test for dichotomous variables and overall classification. The *α*-level was set at *p* ≤ 0.05 for statistical significance. For measures with an occurrence on less than 4 farms, no statistical tests were conducted.

Although AD tests were planned to be conducted at the feeding rack, they needed to be conducted in the barn or from outside a single box for some calves on some farms due to varying housing conditions in calves. To assess the potential effect of test location on AD, a linear mixed model with AD of individual calves as target variable including place of AD test (single housing, group housing at feeding place or in the group housing barn) and age category (0–2 or 3–6 months) as fixed and farm as random factor was used first across all farms (*n* = 20 for ES and n = 20 for CCC farms as test location was not available for the other farms). Because test location had an effect, rearing system (CCC, *n* = 170 calves or ES, *n* = 233 calves) was added as a fixed factor to the model in a second round to test for a potential effect of rearing system on AD. The dependent variable was square-root transformed. Further, a model with the same fixed and random factors was calculated with the proportion of animals that accepted to be touched (AD = 0) per farm and test location as dependent variable. To compare the Criterion score “Good human-animal relationship” in calves, calculations with categories of AD were performed according to the WQP and a regression model including farm, rearing system and percentage of tests in single housing (because only single housing was a predictor for the percentage of animals with AD = 0) was calculated. Model assumptions (normal distribution and homoscedasticity of residuals) were inspected graphically.

## 3 Results

### 3.1 Farms and animals

The 50 dairy farms were distributed across 8 of the 9 Austrian federal states. Herd size on the farm ranged from 10 to 82 cows, 7 to 49 heifers and 2 to 24 calves, without a difference between rearing systems (Table 5). Cow-calf contact and ES farms differed in organic status [CCC: 100%, ES: 56%, Table 5]. Twenty CCC and 16 ES farms used dual purpose breeds for cows, mainly Fleckvieh, further Original Brown Cattle, Pinzgauer, and Grey Cattle. Two CCC and 5 ES farms had crossbreeds of milk x dual purpose (Fleckvieh x Holstein Friesian, Fleckvieh x Red Pied, Jersey mix), 2 CCC and 2 ES farms had both dual purpose and dairy breeds and 1 CCC and 2 ES farms had only dairy breeds (Holstein Friesian, Brown Swiss). Cows whose calves were to be sold for veal or beef production were sometimes crossbred with beef breeds (Limousin, Charolais, Blue-White Belgian). Table 6 shows the distribution of types of CCC across the visited farms.

**Table 5:** Number of animals of different age groups on the visited farms.

No. animals/no. organic farms	CCC^1^ mean ± SD, range	ES mean ± SD, range
No. calves per farm	9.3 ± 5.21, 2–23	11.8 ± 5.83, 3–24
No. heifers per farm	17.1 ± 7.96, 7–36	20.8 ± 10.75, 4–49
No. cows per farm	29.8 ± 17.91, 10–82	35.0 ± 13.54, 14–63
No. organic farms	25	14

^1^On 6 CCC farms, no heifers were reared, so data only from 19 farms available.

**Table 6:** No. of CCC farms (*n* = 25) with each type and daily duration of CCC according to (46).

Type of CCC	Whole-Day	Half-day	2-3x	Total
Dam	8	1	4	13
Foster cow	4	–	3	7
Mix	2	–	3	5
Total	13	1	11	25

### 3.2 Measures

#### 3.2.1 Behavior

##### 3.2.1.1 Avoidance distance

Table 7 shows farm values of AD (median and percentage of animals in distance categories according to the WQP). Models for AD in calves showed an effect of test location (F_2,396_ = 24.913, *p* < 0.001) with AD being lowest for single boxes, slightly higher for grouped-housed animals tested at the feeding place and highest when tested in barn. There was no effect of rearing system on AD in calves (F_1,44_ = 1.295, *p* = 0.261, back-transformed estimated means CCC: 27.6 cm, *n* = 170 calves; ES: 18.8 cm, *n* = 233 calves) nor of age category. The model with the proportion of animals that accepted touch (AD = 0) also revealed an effect of location (F_2,31_ = 5.752, *p* = 0.007) but no effect of rearing system (F_1,47_ = 0.001, *p* = 0.976, estimated means CCC: 33.7%; ES: 34.0%) nor age category. Farm had a highly significant effect (*p* < 0.001 for AD and *p* = 0.002 for AD = 0). There was no influence of rearing system on median AD in heifers (Table 7).

**Table 7:** Results for avoidance distance (AD).

Avoidance distance (AD)^1^	CCC Calves *n* = 25 farms	ES Calves *n* = 25 farms		CCC Heifers *n* = 19 farms	ES Heifers *n* = 25 farms	
	Median, range	Median, range		Median, range	median, range	*Mann–Whitney U Test*: *p, Z*
Median AD	30, 0–180	10, 0–120		20, 0–45	10, 0–40	0.144, 1.460
% animals with AD = 0 cm	11, 0–100	29, 0–100		27 (0–90)	36 (16–92)	0.063, 1.861
% animals with AD > 0–50 cm	41, 0–100	53, 0–100	^1^	45 (10–88)	44 (8–83)	^1^
% animals with AD > 50–100 cm	14, 0–50	6, 0–29	^1^	16 (0–50)	8 (0–33)	^1^
% animals with AD > 100 cm	11, 0–67	0, 0–71	^1^	0 (0–20)	0 (0–27)	^1^

Descriptive statistics are shown for both calves and heifers, and test results for potential differences between farms with cow-calf-contact (CCC) or early separation (ES) are shown for heifers. For statistics and results in calves: see text (2.4 and 3.2.1.1).

^1^The proportion of animals in this distance category was only analyzed descriptively and used for the calculation of the WQP Criterion score for “Good human-animal relationship”.

##### 3.2.1.2 Qualitative behavior assessment

Scores for QBA were higher for both calves and heifers on CCC compared to ES farms (Table 8).

**Table 8:** Results for qualitative behavior assessment (QBA) and quantitative behavior observation on farms with cow-calf contact (CCC) and early separation (ES).

Measure	CCC Calves *n* = 25 farms	ES Calves *n* = 25 farms	Statistics	CCC Heifers *n* = 19 farms	ES Heifers *n* = 25 farms	Statistics
**QBA**	Mean ± SD median, range	Mean ± SD median, range	*t-test*: p t, df	Mean ± SD median, range	Mean ± SD median, range	*t-test*: p t, df
QBA Score	73 ± 12.9 74, 52–100	54 ± 12.7 53, 28–82	**<0.001** 5.31, 48	60 ± 18.1 58, 31–95	48 ± 13.6 46, 21–70	**0.022** 2.41, 32
**Behavior** [no. events/animal/h]	Median, range (no. farms with frequency > 0)	Median, range (no. farms with frequency > 0)	Mann–Whitney U Test: *p*, *z*	Median, range (no. farms with frequency > 0)	Median, range (no. farms with frequency > 0)	Mann–Whitney U Test: *p*, *z*
**Non-nutritive oral behaviors**	3.1, 0–12.82 (23)	5.1, 0–27.5 (24)	**0.030**, −2.174	0.73, 0–11 (16)	1.9, 0.39–5.63 (25)	0.132, −1.505
*Tongue rolling*	0, 0–4.3 (5)	0, 0–6 (6)		0, 0–10 (5)	0, 0–2.62 (10)	
*Object Licking*	3.1, 0–12.82 (23)	4.8, 0–27.50 (24)		0.7, 0–6.76 (16)	1.6, 0–5.21 (24)	
*Cross-sucking*	0, 0–0.79 (5)	0, 0–1.56 (9)		0, 0–0.43 (3)	0, 0–1.33 (9)	
*Urine drinking*	0, 0–0.57 (2)	0, 0–0.20 (3)		0, 0–0.14 (1)	0, 0–0.4 (4)	
**Agonistic**	0, 0–1.22 (9)	0.1, 0–2 (14)	0.131, −1.511	1.2, 0–5.42 (17)	1.3, 0.1–4.3 (25)	0.868, −0.166
*Head butt*	0, 0–0.4 (4)	0, 0–0.8 (9)		0.7, 0–3 (13)	0.6, 0–3.9 (18)	
*Displacement*	0, 0–1.22 (7)	0, 0–1.20 (10)		0.7, 0–5 (15)	0.7, 0–2.29 (23)	
*Fighting*	0, 0 (0)	0, 0–0.09 (1)		0, 0–0.22 (1)	0, 0–0.13 (2)	
*Chasing*	0, 0–0.14 (1)	0, 0–0.43 (3)		0, 0–0.30 (5)	0, 0–1.33 (4)	
*Chasing up*	0, 0–0.28 (1)	0, 0–0.14 (2)		0, 0–0.42 (2)	0, 0–0.32 (2)	
**Cohesive**	1.1, 0–6.78 (23)	1.1, 0–6.2 (22)	0.587, −0.544	1.8, 0–3.25 (16)	1.2, 0–6.25 (20)	0.905, −0.119
*Social Licking*	*0.9, 0–6.56 (20)*	*1, 0–6.2 (22)*		*0.7, 0–2 (16)*	*0,5, 0–5.63 (20)*	
*Horning*	*0.3, 0–2.61 (15)*	*0, 0–3 (11)*		*0.2, 0–2.25 (12)*	*0.2, 0–1 (17)*	
**Play**	1.3, 0–9.29 (20)	1, 0–6.67 (21)	0.785, −0.272	0.2, 0–3.05 (11)	0.1, 0–1.33 (13)	0.601, 0.523
*Locomotor Play*	*1, 0–9.14 (20)*	*1, 0–6.67 (21)*		*0, 0–2.67 (6)*	*0, 0–0.63 (9)*	
*Mounting*	*0, 0 (7)*	*0, 0–0.7 (7)*		*0, 0–0.92 (7)*	*0, 0–1.33 (11)*	
**Health related**	0.7, 0–9.4 (16)	1.7, 0–7.67 (21)	0.221, −1.224	0.4, 0–3.62	0.7, 0–5.11	0.335, −0.964
*Sneezing*	*0, 0–0.50 (4)*	*0, 0–1 (4)*		*0, 0 (0)*	*0, 0–0.31 (4)*	
*Coughing*	*0.6, 0–9.4 (15)*	*1.2, 0–6.68 (21)*		*0.4, 0–3.62 (13)*	*0.7, 0–4.96 (20)*	
*Falling*	*0, 0 (0)*	*0, 0 (0)*		*0, 0–0.25 (1)*	*0, 0–0.89 (1)*	

Observed behaviors were grouped into non-nutritive oral behaviors, agonistic, cohesive, play and health related behaviors. For QBA, descriptive statistics and results of statistical tests (t-tests) are shown. For results of the quantitative behavior observation, descriptive statistics for every behavior are shown, and statistical tests (Mann–Whitney U Tests) were only performed for grouped behaviors. Grouped behaviors and significant *p*-values are (*p* ≤ 0.05) are marked in bold, single observed behaviors are marked in italics.

##### 3.2.1.3 Quantitative behavior observation

Cow-calf-contact calves showed a lower frequency of non-nutritive oral behaviors (Table 8). This variable is largely influenced by object licking (Table 8), which occurred on nearly all farms (CCC: 23, ES: 24 farms). In contrast, the other three non-nutritive oral behaviors were observed on at most 30% of the farms, i.e., cross-sucking on 5 CCC and 9 ES, tongue rolling on 5 CCC and 6 ES, and urine drinking on 2 CCC and 3 ES farms (Table 8). No other observed behaviors, i.e., social, play or health-related, differed between CCC and ES farms (Table 8). No differences were found for heifers (Table 8).

#### 3.2.2 Physical parameters

Cow-calf contact calves were less often affected by lesions and CCC heifers had overgrown claws less often than those on ES farms (Table 4). No differences were found for all other physical indicators and for mortality (Table 4).

#### 3.2.3 Resource- and management-based measures

Cow-calf contact calves had access to a sufficient number of clean and functioning waterpoints more often than ES calves (Table 4). Both CCC calves and heifers had more space and more days with longer than 6 h of access to pasture (Table 4). This difference was still significant when only considering organic farms (Table 4). Moreover, CCC calves and heifers were disbudded less often than those from ES farms (Table 4). All but two ES farms fed their calves restrictively after the first week of life (Table 9).

**Table 9:** Average milk allowance/day in week 1 and maximum milk allowance/day on the participating ES farms (*n* = 25).

	Week 1^1^	Maximum^2^
6 L or less	10	3
> 6–8 L	7	6
> 8–10 L	0	6
> 10–12 l	0	6
> 12 or ad libitum	5	2
No. meals		
2	15	20
3	5	2
4	1	0
Ad libitum	1	1

Number of farms providing the respective milk allowance are given.

^1^Only data from 22 out of 25 farms available.

^2^Only data from 23 out of 25 farms available.

### 3.3 Criterion and principle scores

Criterion and Principle scores are summarized in Table 10. The model for “Good human-animal relationship” in calves (*R*^2^ = 0.063, *F* = 1.025, *p* = 0.390) revealed no influence of rearing system (regression coefficient *B* = 8.326, *p* = 0.308, Table 10). Both CCC calves and heifers scored higher in the Criteria “Ease of movement”, “Absence of pain induced by management procedures”, “Other behaviors” (i.e. access to pasture) and “Positive emotional state” (i.e. QBA, (Table 10). No differences in the other Criterion scores were found for calves and heifers (Table 10). Cow-calf contact calves had higher Principle scores for “Good feeding”, “Good housing” and “Appropriate Behavior” (Table 10).

**Table 10:** Criterion and Principle scores for calves and heifers.

	**CCC Calves** n = 25 farms	**ES Calves** n = 25 farms		**CCC Heifers** n = 19 farms	**ES Heifers** n = 25 farms	
**Criterion**	mean ± SD median, range	mean ± SD median, range	*Mann-Whitney U Test:* **p, z**	mean ± SD median, range	mean ± SD median, range	*Mann-Whitney U Test:* **p, z**
Absence of prolonged hunger^1^	93 ± 27 100, 3-100	89 ± 31 100, 5-100	0.696 0.391	81 ± 34 100, 7-100	87 ± 31 100, 11-100	0.491 0.689
Absence of prolonged thirst^1^	68 ± 22 53, 53-100	54 ± 17 53, 20-100	**0.016** 2.400	68 ± 22 53, 53-100	61 ± 21 53, 20-100	0.298 1.040
Comfort around resting^2^	92 ± 23 100, 11-100	91 ± 20 100, 25-100	0.533 0.623	72 ± 28 86, 31-100	69 ± 29 71, 10-100	0.734 0.340
Ease of movement^2^	88 ± 24 100, 0-100	59 ± 29 52, 9-100	**<0.001** 3.544	94 ± 18 100, 28-100	90 ± 18 99, 23-100	**0.031** 2.159
Absence of injuries^3^	97 ± 4 100, 80-100	92 ± 11 97, 53-100	0.107 1.614	72 ± 19 73, 44-96	64 ± 21 64, 33-100	0.132 1.505
Absence of disease^3^	59 ± 33 55, 22-100	53 ± 27 45, 12-100	0.880 0.151	71 ± 24 100, 29-100	79 ± 26 100, 29-100	0.834 0.210
Absence of pain induced by management^3^	88 ± 13 100, 75-100	78 ± 8 75, 75-100	**0.005** 3.001	89 ± 13 100, 75-100	78 ± 8 75, 75-100	**0.003** 3.201
Social behaviour^4^	92 ± 13 100, 58-100	88 ± 14 92, 55-100	0.118 1.563	56 ± 29 57, 8-100	53 ± 22 52, 21-95	0.767 0.296
Other behaviours^4^	47 ± 28 48, 0-82	19 ± 21 0, 0-62	**<0.001** 3.536	79 ± 11 77, 65-100	47 ± 29 55, 0-80	**<0.001** 4.454
Positive emotional state^4^	73 ± 13 74, 52-100	54 ± 13 53, 28-82	**<0.001** 4.356	60 ± 18 58, 31-95	48 ± 14 46, 21-70	**0.037** 2.087
Good human-animal relationship^4,5^	60 ± 26 69, 16-100	71 ± 19 76, 14-100	0.308^5^	66 ± 14 69, 46-97	74 ± 13 78, 48-98	0.056 1.908
**Principle**						
Good feeding	66 ± 23 57, 16-100	53 ± 20 57, 17-100	**0.047** 1.988	63 ± 25 57, 26-100	58 ± 22 57, 22-100	0.712 0.369
Good housing	87 ± 21 99, 22-100	64 ± 24 61, 19-100	**<0.001** 3.364	75 ± 25 68, 32-100	72 ± 25 77, 18-100	0.556 0.588
Good health	64 ± 24 58, 32-100	58 ± 18 54, 28-98	0.356 0.923	70 ± 15 64, 38-94	64 ± 17 59, 37-100	0.121 1.552
Appropriate Behaviour	53 ± 16 53, 23-78	41 ± 13 38, 25-67	**0.011** 2.532	54 ±13 54, 30-78	46 ± 14 47, 19-71	**0.043** 2.026

1 Criterion contributes to Principle “Good feeding”. ^2^Criterion contributes to Principle “Good housing”. ^3^Criterion contributes to Principle “Good health”. ^4^Criterion contributes to Principle “Appropriate behaviour”. ^5^To compare scores for “Good human-animal relationship” in calves a regression model with farm, rearing system and percentage of tests in single housing was calculated to take into account differing test location.

### 3.4 Overall classification

**Figure 1:**
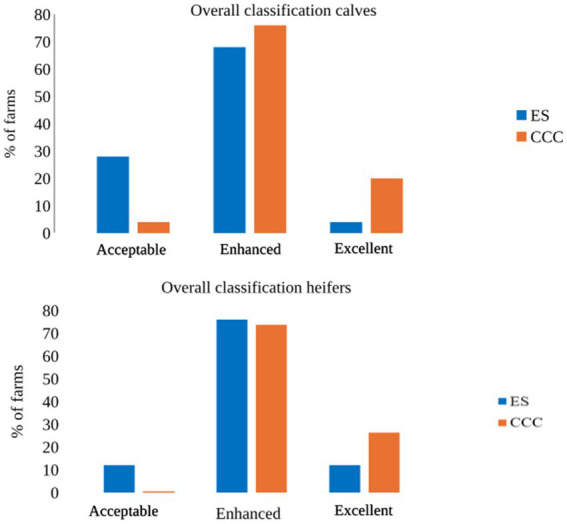
Percentage of visited farms with each overall classification according to the WQP for calves (top) and heifers (bottom). Calves: CCC: *n* = 25, ES: *n* = 25. Heifers: CCC: *n* = 19, ES: *n* = 25.

Figure 1 shows the distribution of farms over the four categories of classifications according to the WQP. Cow-calf contact farms had better overall classification than ES farms for both calves and heifers (Figure 1). Five (= 20 %) CCC farms received an overall classification “excellent” for calves, while this was the case for no ES farm (*p* = 0.023, standardized residuum: |1.6|, Figure 1). Conversely, 1 (4%) CCC and 5 (20%) ES farms were classified as “acceptable” (standardized residuum: |1.5|, Figure 1). For heifers, 5 (26%) CCC and 3 (12%) ES farms scored “excellent” (standardized residuum: CCC: 1.4, ES: −1.2), while 0 CCC and 3 (12%) ES farms scored “acceptable” (standardized residuum: CCC: −1.1, ES: 1.0, Figure 1). No farms in our sample were given the lowest classification “not classified.”

## 4 Discussion

The results of this study confirm our hypothesis of better welfare of calves and heifers on CCC compared to ES farms. This was mainly the case in the dimensions of behavior and mental states including resource- and management-based measures, while there was only a limited effect in the physical dimension. Specifically, CCC calves and heifers showed higher scores for positive emotional state (QBA), had more space, more access to pasture and were less often disbudded compared to ES calves and heifers. In addition, CCC calves showed a lower frequency of non-nutritive oral behaviors, had lesions only on one CCC farm and, in contrast to those on ES farms, were never single housed except for one farm post-weaning. Fewer CCC farms had heifers with overgrown claws. The results led to higher WQ scores for CCC farms in five of the 11 Criteria for calves, four of the 11 Criteria for heifers, three of the four Principles for calves and one of the four Principles for heifers and a higher overall classification for calves for CCC farms. The results thus also confirm our hypothesis that benefits of CCC rearing are more pronounced in calves compared to heifers.

### 4.1 Measures

#### 4.1.1 Behavior

##### 4.1.1.1 Avoidance distance

In line with our hypothesis and with the results of two recent experimental studies (27, 29), there was no difference in AD between rearing systems. In contrast, CCC calves had higher ADs at 1 month of age and tended to do so 1 year later as heifers in a previous study when keeping human-animal interactions to the minimum necessary in the first 4 weeks of life (28); however, the higher the amount of contact during the first week of life, the lower was AD also in CCC calves (28). The amount and quality of human-animal interactions (HAI) determine the AHR (50, 125, 126), reviewed in Waiblinger (59)]. Our results suggest that HAI do not inherently differ between rearing systems. As farmers’ attitudes play the crucial role for HAI [(60); reviewed in Hemsworth and Coleman (61)], farmers valuing a good AHR will engage in regular and positive contact with the calves, independent of rearing system (62–64). Moreover, almost half of the CCC farms had restricted contact where regular interactions between farmers and calves took place during suckling times.

##### 4.1.1.2 Qualitative behavior assessment

The higher QBA (i.e., positive emotional state) scores for both CCC calves and heifers compared to ES indicate a higher emotional well-being in CCC animals. This is in line with previous findings where CCC calves showed longer play behavior as an indicator for positive emotional experiences (19) or a lower stress response to isolation (23) than calves without CCC. Several factors may contribute to this result. First, non-weaned CCC calves receive maternal care from their dam or, even if partly reduced, foster cow (65, 66). The mother is the main social partner during the first weeks of life and CCC calves prefer affiliative interactions with their dam over those with other cows or calves (67). Second, they can suckle a cow, allowing them to (better) fulfil their need to perform sucking behavior (see 4.1.1.3). Suckling an udder leads to higher oxytocin release (16, 68) with anti-stress and other beneficial effects including social bonding (17, 69). Un-weaned calves were also fed restrictively on most ES farms while CCC calves on most farms had a more sufficient milk supply, especially in whole-day systems. Moreover, CCC calves were single-housed only once (and only after weaning), in comparison to those on 15 out of the 25 ES farms, and both CCC calves and heifers had more space than those from ES farms. Social partners are important factors for positive emotional states (70) and space allowance is an important factor for play, a behavior especially important in young animals (71, 72).

##### 4.1.1.3 Quantitative behavior observation

The CCC calves in our study showed fewer non-nutritive oral behaviors, which is consistent with results of previous studies [e.g., (10)]. This is due to a lower occurrence of object licking, what aligns with former findings (73, 74). Non-nutritive oral behaviors such as object licking and cross-sucking are seen as a coping mechanism that could reflect insufficient milk intake, unsatisfied need for suckling, lack of fiber, nutrient deficiencies, frustration, and/or boredom through insufficient environmental enrichment and can become a chronic behavioral disorder with possible negative consequences over long periods of time (9, 11, 75–78). In the present case, it is likely that the better fulfilled sucking motivation through CCC contributed to the lower expression of non-nutritive oral behaviors. Moreover, calves were housed with full-time contact in a cow-calf group on more than half of the CCC farms. This provides a more complex social and physical environment - even compared to calves being housed in same-age groups - which better resembles natural conditions and can lead to additional benefits (19, 79). In contrast, most ES farms fed their calves restrictively and most un-weaned calves were single-housed.

In opposition to other studies [e.g., (12, 27)], cross-sucking occurred on both CCC and ES farms. It was observed on 36% of the ES farms, which is much lower compared to previous studies on Austrian dairy farms with ES (9). However, Größbacher et al. (9) included only group-housed calves, while calves were single-housed on 60% of our ES farms, at least during their first weeks of life. This may have contributed to the differences in prevalences, as single-housing strongly limits or even prohibits (physical) social contact and thus cross-sucking. We observed cross-sucking on 20% of CCC farms in our study. Notably, all of these farms practiced restricted contact to either the dam (2 farms) or a foster cow (1 farm) or mixed (2 farms). This is in line with a previous experimental study where calves in full-time contact systems did not show any cross-sucking, but one of 15 calves did so in a two-times daily restricted suckling system (13). As hunger enhances sucking motivation in calves (75, 80, 81), it can be assumed that restricted contact did not allow to sufficiently fulfil the calves’ nutritive and/or suckling needs. In addition, foster cow systems can pose a risk for foster calves to experience unfulfilled need for sucking and hunger as they may not be allowed to suckle the cow as much as own calves, reflected in lower weight gains (82, 83). This emphasizes potential animal welfare concerns in foster cow systems that should be considered in practice and in future research. It also aligns with consumer perceptions who rate foster cow systems more negative compared to dam-calf contact systems (84).

Absolute values of object licking must be interpreted with caution, as a bout was not required to last for 5 s to be counted as was the case for tongue rolling or cross-sucking (43), potentially inflating this value. Short lickings may rather be seen as a form of explorative behavior (85).

There was no difference in agonistic or affiliative social behavior for both calves and heifers. This is in line with experimental studies comparing CCC and ES animals after weaning and separation that did not find differences in the agonistic interactions that were included in our observations, i.e., displacement and head butts with physical contact (24, 25). In contrast, CCC calves in a whole-day dam-calf-contact system experienced agonistic interactions, both being initiator and receiver, more often than ES calves before weaning (28). However, interaction partners in this study were primarily cows, and it was not differentiated between threats, head butts, avoiding, and submission, so results cannot be compared. In terms of affiliative social behavior, our results confirm previous studies where no difference between CCC or ES calves before or after weaning was found (24, 28).

Despite previous evidence showing that higher space allowance and social companionship increase locomotor play in calves (72), that energy intake affects calves’ play behavior (86–88), and that CCC calves showed higher locomotor play than ES in an experimental study (28), play behavior in our study did not differ between rearing systems. This could be explained because play is considered a relatively rare event and thus repeatability is assumed to be low in case of short observation time (89). Moreover, the proportion of almost half of the CCC farms being farms with restricted contact (Table 6), resulting in temporary restricted milk intake for those calves (see 4.1.1.3), may also have affected the amount of play behavior shown by CCC calves.

#### 4.1.2 Physical parameters

Contrary to farmers’ concerns that keeping calves in cow barns might increase the risk of injuries for calves (90), we found that lesions in calves occurred on more (*n* = 6) ES than CCC (*n* = 1) farms, although still in a low occurrence for both rearing systems. This reflects that CCC farmers in our sample took sufficient precautions to prevent their calves from injuries or skin lesions. It is possible that the findings also reflect a lower incidence of skin diseases and/or faster healing in CCC calves that may result from a better immune response compared to ES calves, possibly due to higher oxytocin levels (16, 17). However, there is only limited evidence on the influence of cow-contact on the calf’s immune system (6).

The lack of differences in the other physical parameters is in line with previous studies that showed conflicting effects of CCC on calf health [(27, 29); reviewed in Beaver et al. (6)]. On the one hand, contact to a cow has the potential to enhance calf health through increased milk intake and potential benefits on the immune system, while on the other hand, infectious pressure might be higher when calves are kept in the same environment as (a) cow(s), which can increase the risk for diseases [reviewed in Beaver et al. (6)]. Moreover, in CCC systems, close observation of the calf’s colostrum intake is necessary to evaluate the amount and be able to assist if necessary, as increased risk of failure of passive immune transfer has been reported in calves left to suckle their dam (91, 92). However, in a more recent study, the amount of failure of passive immune transfer on CCC farms was similar to the amount in ES farms (93), emphasizing the importance of good colostrum management regardless of rearing system.

Overall, both calves from CCC and ES farms were in good health conditions, as the prevalences of respiratory tract disease, diarrhea and umbilical diseases were lower compared to a recent survey on calf management for Austrian farmers, although mortality was similar (94). However, the results are not completely comparable due to differences in assessment (own observation on 1 day vs. reporting by farmers) and definitions.

That CCC farms had heifers with overgrown claws less often than ES farms might be explained by the finding that CCC farms had more access to pasture, which has been proven to improve claw health (95). However, independent of access to pasture, farmers are responsible for providing adequate claw care. Therefore, the results might also reflect differences in CCC farmers’ attitudes toward animals in their care (see 4.1.3). That no other differences between rearing systems were found for heifers emphasizes the complexity and variety of influencing factors on health beyond contact to a cow during calfhood and confirms our hypothesis.

#### 4.1.3 Resource- and management-based measures

Cow-calf contact calves were always group-housed (except for one case after weaning), had more space and access to a sufficient number of clean and functioning waterpoints more often than those from ES farms. The findings can partly be explained by the fact that CCC calves with unrestricted contact to a cow are automatically group-housed and have access to a higher number of water points (given that they are reachable for calves, what is reflected in the measures as only those drinkers that were accessible for calves were included in the analysis). Moreover, the higher number of group-housed calves on CCC than ES farms could be due to the higher proportion of organic farms among CCC farms, considering that single-housing of calves is not allowed for more than 1 week in organic farming (96). Regardless of rearing system, group-housing is beneficial for social, behavioral, cognitive and physical development and improves animal welfare [(72, 94, 97, 98); reviewed in (70)].

That both CCC calves and heifers had more space and more access to pasture, even when comparing CCC with only organic ES farms as those are required by law to provide their animals with access to pasture (96), aligns with our hypothesis of CCC farmers providing their animals with better management practices. This is in line with the results from previous studies: providing their animals with a more natural environment that enables extensive possibilities for species-specific behavior, a core aspect of organic farming also recognized in the EU Organic legislation, is an important value for many farmers practicing CCC, making them more likely to oppose unnatural practices including ES (32, 96, 99). This aligns with the argumentation that ES can be considered as a violation of the principle of fairness, one of the four principles of organic agriculture (100, 101) and fits with our and the findings of others that CCC is mainly practiced on organic farms (102). Moreover, providing animals with the opportunity to engage in natural behaviors they are strongly motivated to perform, such as going on pasture (103) and CCC (104), have been emphasized as an important aspect also in a broader context of sustainability (38). As CCC and ES farms were balanced for geographical regions, location of the farm should not have influenced the amount of pasture provided. This suggests that for CCC farmers, access to pasture might not only be seen as a necessary aspect to maintain organic status but also as an important factor to fulfil the animals’ behavioral needs.

Fewer CCC (48 % of the CCC farms) than ES farms (88 % of the ES farms) disbudded their calves (one CCC and one ES farm used polled genetics). Keeping intact animals is thus more prevalent in the CCC farms in our study than reported in a European-wide survey, as 80.7% of dairy farms in the EU and 61.2% of cattle farms in general (dairy, beef and suckler together) in Central Europe reported to disbud their animals (105). As keeping intact cattle was least prevalent in dairy farms and loose-housing systems (105), the actual difference can be expected to be even more pronounced. In Austria, it is legally required to use anesthetics and analgesics for disbudding and dehorning of cattle. Although this can prevent acute pain during and shortly after the procedure if applied correctly, it does not necessarily alleviate adverse longer-term effects of disbudding, e.g., wound healing disturbances, formation of neuromata and/or chronically painful states [(106); reviewed in Knierim et al. (107)]. Apart from pain due to the procedure (a major argument against disbudding), horns play an important role in the social behavior of cattle, as horned herds engage in fewer physical agonistic interactions (108), indicating a more stable social structure [reviewed in Menke et al. (107), Knierim et al. (109)]. Moreover, it has been argued that keeping hornless cattle is a violation of the animal’s bodily integrity and is therefore not acceptable from an ethical point of view (109, 110). Improved management, housing conditions, and human-animal relationship are important influential factors for minimizing the risk of keeping horned cattle [e.g., for injuries in animals or humans, (111, 112)]. That CCC farmers disbud their animals less often is in line with the above-mentioned theory that it is important for them to allow their animals a life more aligned with their natural characteristics rather than to follow conventional procedures that make the animal fit better to the environment by altering their natural characteristics. Moreover, higher social competence in CCC animals, especially in whole-day full contact systems (24, 25), may have beneficial effects on social dynamics in a cow herd and ease the keeping of intact cattle with horns.

### 4.2 Criterion and principle scores

#### 4.2.1 Good feeding

The higher WQ score for “Good feeding” in CCC compared to ES calves mainly stems from the difference in the Principle “Absence of prolonged thirst” that reflects the condition of drinkers (4.1.3). Cow-calf contact farms more often scored 100 in this Principle (i.e., having at least 2 clean drinkers available per animal) and never scored below 53 (i.e., less than 2 drinkers available per animal). The latter was the case twice on ES farms, where the one drinker they provided per group was dirty and therefore no sufficient supply of clean water was provided.

The score for “Absence of prolonged hunger,” determined by the percentage of lean animals, did not differ between rearing systems in our study, suggesting that most calves were in an acceptable body condition. However, there are consequences of common restrictive feeding practices in dairy farming beyond extreme malnourishment. Calves received *ad libitum* or a maximum amount of milk of >12 L, coming close to 20% of body weight recommended at least in the first 4 weeks of life (2), on only two out of the 25 ES farms. This corresponds with a recent Austrian-wide questionnaire on calf management (94), where 15.4% of the respondents reported that they fed their calves *ad libitum* milk. Restricted feeding is in contrast to the calves’ natural behavior and can, in addition to being a risk factor for developing non-nutritive oral behaviors (4.1.1.3), lead to less play behavior suggesting lower emotional well-being (87, 113–117).

#### 4.2.2 Good housing

That CCC calves had more space than ES calves is reflected in the higher score for the Criterion “Ease of movement” that contributes most to the difference within the Principle of “Good housing.” The higher space allowance of CCC heifers compared to ES heifers is reflected in the Criterion score for “Ease of movement” but not in the Principle score for “Good housing”, as the other measures balanced the differences between rearing systems. However, the modified calculation of this score (see 2.3) must be considered as a limitation and future projects should aim to establish suitable calculation procedures for dairy calves and heifers.

#### 4.2.3 Good health

As the majority of calves and heifers were in good clinical condition, there was no difference between rearing system in the Principle score for “Good health.” The variation in the Criterion “Absence of pain induced by management decisions” arises from the fact that more of the visited CCC farms do not disbud or dehorn their animal. As tail docking in dairy cattle is prohibited by Austrian law, none of the visited farms followed this practice.

#### 4.2.4 Appropriate behavior

Both CCC calves and heifers scored higher in the Principle “Appropriate Behavior,” which was driven by higher scores for the Criteria “Other behaviors” and “Positive emotional state.” This is in line with our hypothesis, as the main differences were expected in the behavioral dimension. Cow-contact and higher space allowance have been discussed as important factors for a positive emotional state (4.1.1.2), while farmers’ attitudes are an important aspect determining management decisions such as the amount of access to pasture (4.1.1.3). Since animals’ needs can primarily be recognized by their behavior, higher scores in this Principle may indicate that CCC farmers place greater emphasis on meeting animals’ natural behavioral needs.

### 4.3 Overall classification

Differences between rearing systems occurred not only in single measures but also in the aggregated overall classification for calves, pointing towards enhanced welfare states in CCC compared to ES calves in our sample, with at least one quarter of CCC farms classified as “excellent” and only one farm “acceptable”. Also for heifers, differences point towards better overall well-being in CCC animals (26% of the farms reached “excellent” compared to 12% of the ES farms, and no CCC farm was classified as “acceptable” compared to 12% of the ES farms), although differences were not confirmed statistically. Previous studies conducted in Europe found that no (dairy cows) or single (fattening bulls) farms achieved the classification “excellent,” but 32 to 66% were classified “acceptable” (41, 42, 118, 119). Even though results are not fully comparable due to the assessment of different age groups and slight differences in measures and calculations, it indicates relatively good welfare in the animals on quasi all CCC farms included in our sample.

### 4.4 General discussion and limitations

There was a high variation in the types of CCC practiced by the visited farms. This heterogeneity reflects the situation in practice, as CCC rearing is still rare in commercial dairy farming and CCC farmers adopt individual solutions. The distribution of farms toward the different systems fits well with an earlier online-survey among Austrian farmers (120) and has also been recognized in studies from other countries (32, 121). Thus, our sample can be seen as representative for CCC farms in Austria and has high external validity with respect to CCC systems as a whole. However, our sample does not allow to draw firm conclusions on different types of CCC due to the low sample size per system, although some hints regarding differences in line with previous studies arose (e.g., differences in whole-time dam-contact and short-time systems). Comparability of heifers between the two rearing systems is limited as the data was only available for 19 CCC as opposed to 25 ES farms. Future research should include more CCC farms of the same type to allow conclusions about different CCC systems.

In contrast, ES farms were not a representative sample. There is a bias toward organic farms for ES, as the present sample lies above the Austrian average of 26% for dairy farms (49). Moreover, by convenience sampling, participants may be biased toward being more engaged, motivated and open for science than on average. Therefore, it can be assumed that the welfare of ES animals in the present sample is better than on average.

As it was impossible to blind the assessors due to on-farm assessments, an observer bias (expectation bias) cannot be excluded (122). However, the risk of bias was minimized through standardized, professional training of the assessors by a delegate of the WQ network and adhering to pre-defined definitions. The large variation within farms of both rearing systems with clear overlaps suggests a successful reduction of expectation bias. Besides by training, a potential effect of the observer on differences was minimized because the majority of assessments (38 farms = 76% farms) was done by one assessor, and another assessor evaluated 5 CCC and ES farm each, therefore balancing the two rearing systems nearly perfectly between assessors.

We performed quite a substantial number of statistical tests that altogether may be perceived as multiple testing in a first glance. However, we mainly tested specific, pre-defined hypotheses on single variables. The exception is health where we tested several measures. Thus, the difference regarding lesions in calves and overgrown claws in heifers need to be interpreted with caution but should be investigated further. Regarding WQ criteria and principles scores, the overall picture in calves clearly supports the conclusion of better welfare in CCC calves (with nearly half of the scores differing statistically and other pointing nearly all in the expected direction), but this is less so for heifers, as discussed above already.

As there are currently no calculation formulae for WQ Scores included in the WQP for dairy calves and heifers, the calculations from the WQP for fattening bulls were used. While most of the indicators are observed in both protocols, thresholds for calculations may differ due to age and gender of the animals. In addition, not all the measures proposed to observe in dairy calves and heifers are included in the WQP for fattening bulls, so these measures were not included in the calculation of WQ scores. Future research should aim to establish specific calculations for the WQP for dairy calves and heifers.

The WQP has been developed including stakeholders, aiming for a classification that can be achieved in practice, and animal welfare evaluation is biased due to being conducted from a human point of view (123). This brings the concern that the animals’ perspective may not be sufficiently taken into consideration (123). Moreover, the overall classification in the WQP is based on results on herd level and may not sufficiently consider individual animals (124). Therefore, terms like “acceptable,” “enhanced” or “excellent,” as proposed in the WQP, should be used with caution when describing welfare. Existing problems on herd as well as individual level, regardless of classification status, should be reported to and improved by the farmer.

## 5 Conclusion

Our results point to some benefits for CCC animals in terms of behavior, health and management. While the fact of being reared in contact with a cow is one important factor, other aspects also positively influenced the welfare of animals, including more access to pasture, more space in the barn and less disbudding. As expected, the influence on calf welfare was more pronounced than on heifer welfare, possibly due to the direct contact with a cow and the generally higher vulnerability of calves at a young age. However, the results also pointed toward slight benefits for heifers that were mainly rooted in resources and management parameters, but also included higher QBA scores suggesting higher emotional well-being. Future research should aim to disentangle the effects of different types of CCC on animal welfare on-farm and establish formulae for the calculation of WQ Scores for the WQP for dairy calves and heifers. More holistic farming practices prioritizing animal welfare including CCC rearing should be aimed for in the transition toward more sustainable farming.

## Data Availability

The original contributions presented in the study are included in the article/supplementary material, further inquiries can be directed to the corresponding author.
