# Correction: Welfare of calves and heifers on dairy farms with cow-calf contact rearing or early separation

**DOI:** 10.3389/fvets.2025.1743906

**Published:** 2025-12-09

**Authors:** Anna Rademann, Marie Louise Schneider, Susanne Waiblinger

**Affiliations:** Centre for Animal Nutrition and Welfare, Clinical Department for Farm Animals and Food System Science, University of Veterinary Medicine Vienna, Vienna, Austria

**Keywords:** cattle, dam-calf contact, dairy calves, dairy heifers, behavior, health, welfare assessment, foster cow

In the published article, there was an error in Table 4 as published. The number of farms with disbudding was adapted for calves and heifers as authors discovered that one farm had been classified wrongly, and the number of farms with at least two clean, functioning water points for calves was corrected due to a typo in the previous version (the number of farms equaled 26, although we only visited 25 farms). Moreover, the format of several *z*-values was adapted to standardize it in accordance to the other tables and the odds ratio for the heifer-measures was adapted. The corrected Table 4 and its caption appear below.

**Table 4:** Results for physical and resource/management measures.

	**CCC Calves *n* = 25 farms**	**ES Calves *n* = 25 farms**		**CCC Heifers *n* = 19 farms**	**ES Heifers *n* = 25 farms**	
**Physical parameters**^**1**^ **[%]**	Median, range (no. farms with prevalence >0)	Median, range (no. farms with prevalence >0)	*Mann–Whitney U-test*: **p, z**	Median, range (no. farms with prevalence >0)	Median, range (no. farms with prevalence >0)	*Mann–Whitney U-test*: **p, z**
Hairless patches	0, 0–71 (12)	10, 0–63 (13)	0.828, 0.218	17, 0–87 (18)	22, 0–67 (21)	0.529, 0.629
Diarrhea	0, 0–60 (12)	10, 0–60 (15)	0.302, 1.031	>0 on 20 farms or less, therefore listed below		
Overgrown claws	This measure is not observed in calves			0, 0–30 (5)	4, 0–42 (16)	**0.031**, 2.151
Mortality (last 12 months)	0, 0–24 (11)	0, 0–26 (12)	0.627, 0.486	>0 on 20 farms or less, therefore listed below		
**Physical parameters**^**2**^ **[%]**	Median, range (no. farms with prevalence >0)	Median, range (no. farms with prevalence >0)	*Fisher Exact Test*: ***p*****; OR (95% CI)**	Median, range (no. farms with prevalence >0)	Median, range (no. farms with prevalence >0)	*Fisher Exact Test*: ***p*****; OR (95% CI)**
Lean animals	0, 0–33 (2)	0, 0–25 (3)	1; 0.67 (0.12–3.65)	0, 0–14 (5)	0, 0–10 (4)	0.467; 1.86 (0.41–8.22)
Dirtiness	0, 0–50 (3)	0, 0–33 (5)	0.702; 0.60 (0.16–2.25)	0, 0–75 (8)	0, 0–84 (12)	0.766; 0.78 (0.24–2.62)
Lesions	0, 0–7 (1)	0, 0–33 (6)	**0.049;** 0.14 (0.02–1.01)	0, 0–21 (6)	0, 0–58 (9)	1; 0.82 (0.23–2.91)
Swellings	0, 0 (0)^3^	0, 0–33 (3)^3^	^4^	0, 0–21 (5)	0, 0–58 (6)	0.759; 1.24 (0.35–4.35)
Nasal discharge	0, 0–17 (7)	0, 0–33 (5)	0.742; 1.40 (0.51–3.82)	0, 0–19 (6)	0, 0–50 (6)	0.735; 1.46 (0.39–5.55)
Ocular discharge	0, 0–10 (1)	0, 0–26 (4)	0.349; 0.25 (0.03–2.08)	0, 0 (0)	0, 0–11 (5)	0.060; –
Diarrhea	>0 on more than 20 farms, therefore listed above			0, 0–23 (5)	0, 0–10 (4)	0.467; 1.88 (0.41–8.22)
Hampered respiration	0, 0–11 (1)	0, 0 (0)	^4^	0, 0–3 (1)	0, 0 (0)	^4^
Lameness	0, 0 (0)	0, 0 (0)	^4^	0, 0–8 (1)	0, 0–13 (1)	^4^
Bloated rumen	0, 0 (0)	0, 0 (0)	^4^	0, 0 (0)	0, 0–4 (1)	^4^
Mortality (last 12 months)	>0 on more than 20 farms, therefore listed above			0, 0 (0)	0, 0–4 (2)	^4^
**Resource and management**	Median, range	Median, range	*Mann–Whitney U-test*: **p, z**	Median, range	Median, range	*Mann–Whitney U-test*: **p, z**
Min. space per animal^5^	8.06, 1.35–21.32	2.67, 1.17–11.39	**< 0.001**, 3.949	18.3, 3.8–150	8.8, 3.6–41.4	**< 0.001**, 3.234
No. days >6 h on pasture	103, 0–224	0, 0–138	**< 0.001**, 3.536	192, 150–356	121, 0–210	**< 0.001**, 4.454
No. days >6 h on pasture (only organic)	103, 0–224	61, 0–138	**0.015**, 2.431	192, 150–356	150, 89–210	**0.002**, 3.040
**Resource and management**	No. farms	No. farms	*Fisher Exact Test*: ***p*****; OR (95% CI)**	No. farms	No. farms	*Fisher Exact Test*: ***p*****; OR (95% CI)**
Min. 2 clean, functioning water points: yes/no	8/17	2/23	0.074; 5.41 (0.04–0.98)	6/13	5/20	1; 1.26 (0.33–4.84)
Disbudding: yes/no	12/13	22/3	**0.005**; 7.94 (1.88–33.50)	8/11	22/3	**0.003**; 10.08 (2.22–45.71)

Descriptive statistics and test results for potential differences between farms with cow-calf-contact (CCC) or early separation (ES) are shown. Significant *p*-values (*p* ≤ 0.05) are marked in bold. Ear infections and umbilical infections were never observed and thus are not listed.

^1^Physical parameters with prevalences >0 on more than 20 farms.

^2^Physical parameters with prevalences >0 on 20 farms or less.

For swellings, values for calves from 2 CCC and 2 ES farms were not available.

^4^Due to occurrence on less than four farms, no statistical tests were conducted for these measures.

^5^For calves: m^2^/animal, for heifers: m^2^/700 kg.

^6^For comparison of only organic farms: CCC: *n* = 25 (calves) or *n* = 19 (heifers), ES: *n* = 14 (calves and heifers).

In the published article, there was an error in Table 10 as published. The descriptive and statistical values for Absence of prolonged thirst (only ES calves and CCC heifers), Comfort around resting (only calves), ease of movement (only CCC heifers), Absence of injuries (only ES calves), Absence of disease, Absence of pain induced by management (for both CCC and ES calves and heifers), Good feeding (only ES calves), Good housing (CCC and ES calves and ES heifers) and Good health (both CCC and ES calves and heifers) slightly changed. This is (1) due to the detected changes for the number of clean, functioning water points and disbudding and (2) due to slight necessary corrections for the calculation procedures for ease of movement, absence of injuries and absence of disease that were not displayed correctly in the submitted version. There is now a significant difference for the Criterion score for “Ease of movement” in heifers. However, this does not change the scientific conclusions as the underlying measure “space allowance” had shown a significant difference before and is already discussed in the published manuscript. In addition, there is now a significant difference for “Good feeding” in calves. Also here, this does not change the scientific conclusion as the significant difference in the underlying measure “Number of clean, functioning water points” has been thoroughly discussed in the submitted version. The corrected Table 10 and its caption appear below.

**Table 10:** Criterion and principle scores for calves and heifers.

	**CCC Calves *n* = 25 farms**	**ES Calves *n* = 25 farms**		**CCC Heifers *n* = 19 farms**	**ES Heifers *n* = 25 farms**	
**Criterion**	Mean ± SD median, range	Mean ± SD median, range	*Mann–Whitney U-test:* **p, z**	Mean ± SD median, range	Mean ± SD median, range	*Mann-Whitney U-test:* **p, z**
Absence of prolonged hunger^1^	93 ± 27 100, 3–100	89 ± 31 100, 5–100	0.696 0.391	81 ± 34 100, 7–100	87 ± 31 100, 11–100	0.491 0.689
Absence of prolonged thirst^1^	68 ± 22 53, 53–100	54 ± 17 53, 20–100	**0.016** 2.400	68 ± 22 53, 53–100	61 ± 21 53, 20–100	0.298 1.040
Comfort around resting^2^	92 ± 23 100, 11–100	91 ± 20 100, 25–100	0.533 0.623	72 ± 28 86, 31–100	69 ± 29 71, 10–100	0.734 0.340
Ease of movement^2^	88 ± 24 100, 0–100	59 ± 29 52, 9–100	**< 0.001** 3.544	94 ± 18 100, 28–100	90 ± 18 99, 23–100	**0.031** 2.159
Absence of injuries^3^	97 ± 4 100, 80–100	92 ± 11 97, 53–100	0.107 1.614	72 ± 19 73, 44–96	64 ± 21 64, 33–100	0.132 1.505
Absence of disease^3^	59 ± 33 55, 22–100	53 ± 27 45, 12–100	0.880 0.151	71 ± 24 100, 29–100	79 ± 26 100, 29–100	0.834 0.210
Absence of pain induced by management^3^	88 ± 13 100, 75–100	78 ± 8 75, 75–100	**0.005** 3.001	89 ± 13 100, 75–100	78 ± 8 75, 75–100	**0.003** 3.201
Social behaviour^4^	92 ± 13 100, 58–100	88 ± 14 92, 55–100	0.118 1.563	56 ± 29 57, 8–100	53 ± 22 52, 21–95	0.767 0.296
Other behaviours^4^	47 ± 28 48, 0–82	19 ± 21 0, 0–62	**< 0.001** 3.536	79 ± 11 77, 65–100	47 ± 29 55, 0–80	**< 0.001** 4.454
Positive emotional state^4^	73 ± 13 74, 52–100	54 ± 13 53, 28–82	**< 0.001** 4.356	60 ± 18 58, 31–95	48 ± 14 46, 21–70	**0.037** 2.087
Good human-animal relationship^4, 5^	60 ± 26 69, 16–100	71 ± 19 76, 14–100	0.308^5^	66 ± 14 69, 46–97	74 ± 13 78, 48–98	0.056 1.908
**Principle**						
Good feeding	66 ± 23 57, 16–100	53 ± 20 57, 17–100	**0.047** 1.988	63 ± 25 57, 26–100	58 ± 22 57, 22–100	0.712 0.369
Good housing	87 ± 21 99, 22–100	64 ± 24 61, 19–100	**< 0.001** 3.364	75 ± 25 68, 32–100	72 ± 25 77, 18–100	0.556 0.588
Good health	64 ± 24 58, 32–100	58 ± 18 54, 28–98	0.356 0.923	70 ± 15 64, 38–94	64 ± 17 59, 37–100	0.121 1.552
Appropriate behavior	53 ± 16 53, 23–78	41 ± 13 38, 25–67	**0.011** 2.532	54 ±13 54, 30–78	46 ± 14 47, 19–71	**0.043** 2.026

Descriptive statistics and results of Mann–Whitney *U*-test between CCC (calves: no. farms = 25, heifers: *n* = 19) and ES (*n* = 25) farms or, for “Good human-animal relationship,” regression model's *p*-value, are shown. Significant *p*-values (*p* ≤ 0.05) are marked in bold.

^1^Criterion contributes to Principle “Good feeding.”

^2^Criterion contributes to Principle “Good housing.”

^3^Criterion contributes to Principle “Good health.”

^4^Criterion contributes to Principle “Appropriate behavior.”

^5^To compare scores for “Good human-animal relationship” in calves, a regression model with farm, rearing system and percentage of tests in single housing was calculated to take into account differing test location.

In the published article, there was an error in Figure 1 as published. The number of ES farms scoring “excellent” or “enhanced” was adapted for calves. The corrected Figure 1 and its caption appear below.

**Figure 1:**
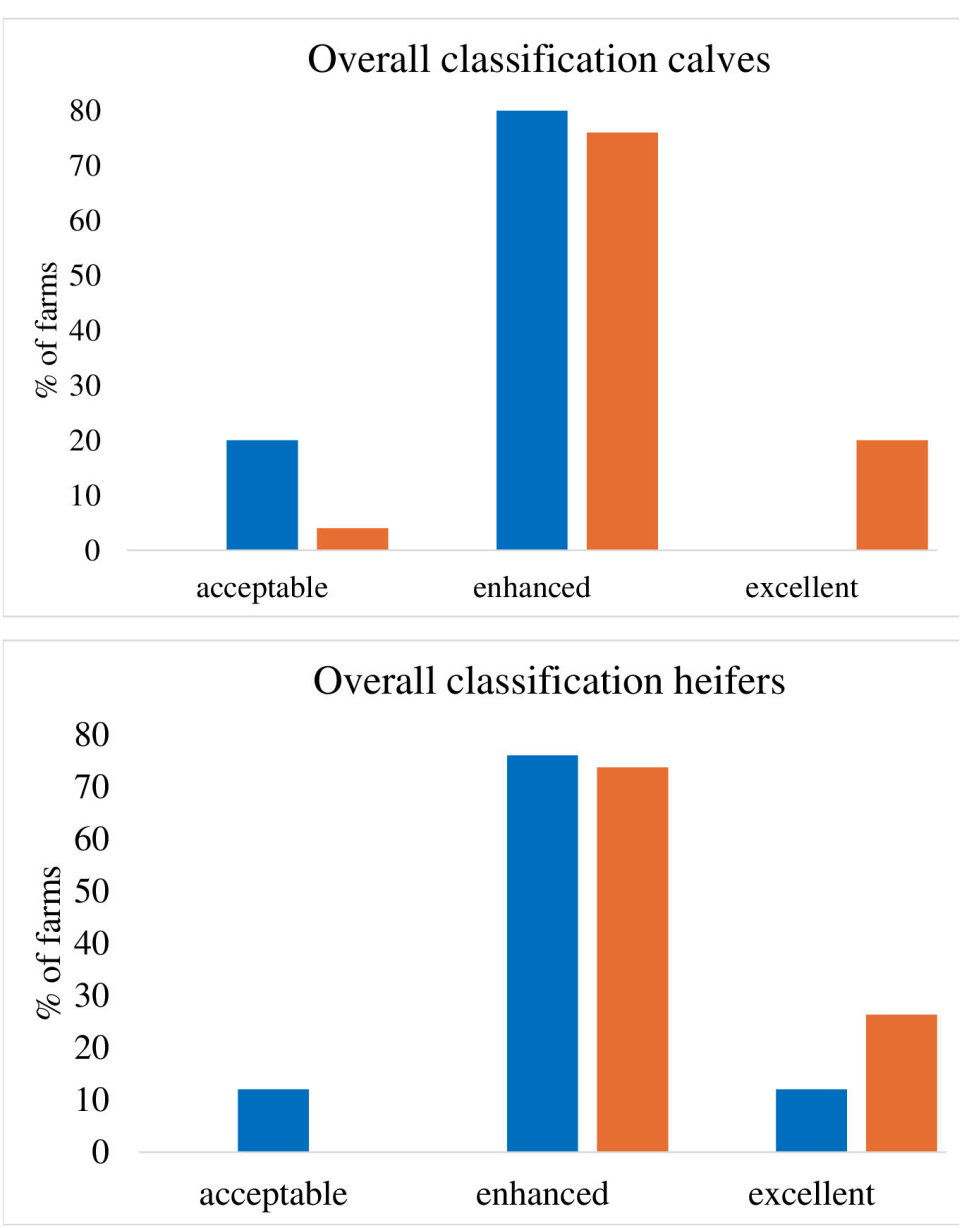
Percentage of visited farms with each overall classification according to the WQP for calves **(top)** and heifers **(bottom)**. Calves: CCC: *n* = 25, ES: *n* = 25. Heifers: CCC: *n* = 19, ES: *n* = 25.

In the published article, there was an error. Resulting from the changes described above, some sections of the text needed to be adapted.

A correction has been made to three sentences in the **Abstract**. The first sentence previously stated:

“Both CCC calves and heifers had more space (calves and heifers: *p* < 0.001), were less often disbudded (calves: *p* = 0.032, heifers: *p* = 0.020) and had more access to pasture (*p* < 0.001).”

The corrected sentence appears below:

“Both CCC calves and heifers had more space (calves and heifers: *p* < 0.001), were less often disbudded (calves: *p* = 0.002, heifers: *p* = 0.003) and had more access to pasture (*p* < 0.001).”

The second sentence previously stated:

“Accordingly, rearing systems differed in Criterion and Principle scores. Both CCC calves (*p* = 0.011) and heifers (*p* = 0.043) scored higher in “Appropriate Behavior” and calves scored higher in “Good housing” (*p* = 0.001).”

The corrected sentence appears below:

“Accordingly, rearing systems differed in Criterion and Principle scores. Both CCC calves (*p* = 0.011) and heifers (*p* = 0.043) scored higher in “Appropriate Behavior” and calves scored higher in “Good feeding” (*p* = 0.047) and “Good housing” (*p* = 0.001).”

The third sentence previously stated:

“CCC farms had a better WQ classification than ES farms (calves: *p* = 0.022), heifers: *p* = 0.046) with 20% or 32% of CCC farms reaching “excellent” for calves or heifers compared to 4 or 8%, respectively.

The corrected sentence appears below:

“CCC farms had a better WQ classification than ES farms for calves (*p* = 0.023), and 20% or 26% of CCC farms reached “excellent” for calves or heifers compared to 0 or 12%, respectively.”

A correction has been made to three sentences in the **Section 3.3 Criterion and Principle Scores**. The first sentence previously stated:

“Both CCC calves and heifers scored higher in the Criteria “Absence of pain induced by management procedures,” “Other behaviors” (i.e., access to pasture), and “Positive emotional state” (i.e., QBA).

The corrected sentence appears below:

“Both CCC calves and heifers scored higher in the Criteria “Ease of movement,” “Absence of pain induced by management procedures,” “Other behaviors” (i.e. access to pasture), and “Positive emotional state” (i.e. QBA, Table 10).”

The second sentence previously stated:

“In addition, calves differed in the Criterion score “Ease of movement” (Table 10).”

This sentence can be deleted, as the information is integrated in the previous sentence.

The third sentence previously stated:

“Cow-calf contact calves had higher Principle scores for “Good housing” and “Appropriate Behavior” (Table 10).”

The corrected sentence appears below:

“Cow-calf contact calves had higher Principle scores for “Good feeding,” “Good housing,” and “Appropriate Behavior” (Table 10).”

A correction has been made to one sentence in the **Section 3.4 Overall classification**. The sentence previously stated:

“Five (= 20%) CCC farms received an overall classification “excellent” for calves, while this was only the case for 1 (4%) ES farm (*p* = 0.034, standardized residuum: |1.2|, Figure 1).”

The corrected sentence appears below:

“Five (= 20%) CCC farms received an overall classification “excellent” for calves, while this was the case for no ES farm (*p* = 0.023, standardized residuum: |1.6|, Figure 1).”

A correction has been made to one sentence in the **Section 4 Discussion**. The sentence previously stated:

“The results led to higher WQ scores for CCC farms in five of the 11 Criteria for calves, three of the 11 Criteria for heifers, two of the four Principles for calves and one of the four Principles for heifers and a higher overall classification for CCC farms for both calves and heifers.”

The corrected sentence appears below:

“The results led to higher WQ scores for CCC farms in five of the 11 Criteria for calves, four of the 11 Criteria for heifers, three of the four Principles for calves and one of the four Principles for heifers and a higher overall classification for calves for CCC farms.”

A correction has been made to one sentence in the **Section 4.1.3 Resource- and management-based measures, Paragraph 3, sentence 1**. The sentence previously stated:

“Fewer CCC (52% of the CCC farms) than ES farms (84% of the ES farms) disbudded their calves (one CCC and one ES farm used polled genetics).”

The corrected sentence appears below:

“Fewer CCC (48% of the CCC farms) than ES farms (88% of the ES farms) disbudded their calves (one CCC and one ES farm used polled genetics).”

A correction has been made to one sentence in the **Section 4.2.2 Good housing**. The sentence previously stated:

“Even though CCC heifers had more space than ES heifers, it was not reflected in the Criterion or Principle score, as the other measures balanced the differences between rearing systems.”

The corrected sentence appears below:

“The higher space allowance of CCC heifers compared to ES heifers is reflected in the Criterion score for “Ease of movement” but not in the Principle score for “Good housing,” as the other measures balanced the differences between rearing systems.”

A correction has been made to **Section 4.3 Overall classification**. The sentence previously stated:

“Differences between rearing systems occurred not only in single measures but also in the aggregated overall classification, pointing toward enhanced welfare states in CCC compared to ES animals in our sample, with at least one quarter of CCC farms classified as “excellent” and only one farm “acceptable” in calves.”

The corrected sentence appears below:

“Differences between rearing systems occurred not only in single measures but also in the aggregated overall classification for calves, pointing toward enhanced welfare states in CCC compared to ES calves in our sample, with at least one quarter of CCC farms classified as “excellent” and only one farm “acceptable.” Also for heifers, differences point toward better overall well-being in CCC animals (26% of the farms reached “excellent” compared to 12% of the ES farms, and no CCC farm was classified as “acceptable” compared to 12% of the ES farms), although differences were not confirmed statistically.”

The original article has been updated.

